# Isolated short stature as the only presenting symptom of glycogen storage disease type 0a in a Chinese child: A case report

**DOI:** 10.1097/MD.0000000000039091

**Published:** 2024-08-09

**Authors:** Hao Fu, Aoyu Yang, Caiqi Du, Yan Liang

**Affiliations:** a Department of Pediatrics, Tongji Hospital, Tongji Medical College, Huazhong University of Science and Technology, Wuhan, China.

**Keywords:** continuous glucose monitoring, glycogen storage disease type 0a, short stature, uncooked cornstarch

## Abstract

**Rationale:**

Glycogen storage disease type 0a (GSD0a) is a rare autosomal recessive disorder caused by glycogen synthase deficiency. Short stature is a characteristic feature in 29% of GSD0a patients, but isolated short stature as the only presenting symptom is exceedingly rare, with only 2 cases reported worldwide.

**Patient concerns:**

A 4-year-old girl presented with persistent growth retardation despite previous treatment for renal tubular acidosis.

**Diagnoses:**

Based on clinical presentation and whole exome sequencing results, the patient was diagnosed with GSD0a.

**Interventions:**

Uncooked cornstarch therapy was initiated at 2 g/kg every 6 hours.

**Outcomes:**

After 3 years of treatment, the patient’s height SDS improved from −2.24 to −1.06, with enhanced glycemic control and no complications.

**Lessons:**

This case emphasizes considering GSD0a in unexplained short stature and the value of continuous glucose monitoring. Early diagnosis and treatment can optimize growth in GSD0a patients.

## 1. Introduction

Glycogen storage disease type 0a (GSD0a, OMIM#240600) is an autosomal recessive disorder caused by a deficiency in glycogen synthase (GS), encoded by the *GYS2* gene. The clinical manifestations of GSD0a are diverse, with the most common initial symptoms being nausea, vomiting, and pallor. In severe cases, patients may present with neuropsychiatric symptoms such as somnolence, altered consciousness, and seizures. Biochemical investigations can reveal fasting ketotic hypoglycemia, postprandial hyperglycemia, and hyperlactatemia. Approximately 41% of cases are asymptomatic and are often identified through familial screening.^[1]^ Short stature is another characteristic feature, affecting about 29% of GSD0a patients.^[1]^ However, the presentation of isolated short stature as the initial symptom is exceedingly rare, with only 2 cases documented worldwide to date.^[2,3]^

Here, we report the first Chinese case of GSD0a presenting with isolated short stature as the sole presenting symptom. This case highlights the importance of early diagnosis and intervention to optimize the management of patients with GSD0a.

## 2. Case presentation

A 4-year-old girl presented to our hospital with a history of growth retardation for over a year. One year prior, she had been diagnosed with renal tubular acidosis at another hospital due to her short stature and growth delay. She was treated with calcitriol, calcium carbonate D3, and potassium citrate, resulting in a growth rate of 5.6 cm/year; however, detailed information about her treatment was unavailable. To exclude inherited metabolic disorders, the patient underwent blood amino acid and urine organic acid testing at an external facility, which revealed hyperlactatemia and ketosis-associated changes in urine organic acids. Seeking further evaluation and management, the patient presented to our hospital.

The patient was born at full term via vaginal delivery, with a birth weight of 3410 g and a length of 50.0 cm. She was breastfed, but the timing of complementary food introduction is unknown. Her developmental milestones were normal, with head control at 3 months, sitting independently at 6 months, and stable walking at 12 months. Throughout the course of her disease, the patient maintained good mental status, appetite, and sleep, with no reported psychiatric symptoms. The patient’s parents are healthy, and there is no history of consanguinity or genetic disorders in the family.

On examination, the patient’s height was 94.7 cm (<3rd percentile, −2.13 SDS), and her weight was 15.0 kg (25th–50th percentile), with a BMI of 16.7, indicating proportionate short stature. She had no craniofacial or body dysmorphic characteristics. Breast and public hair development were Tanner stage I. Cardiovascular and pulmonary examinations were unremarkable. The abdomen was not distended, and the liver and spleen were not palpable below the costal margin. Physiological reflexes were present, and no pathological reflexes were elicited.

Liver and kidney function tests, electrolytes, and blood gas analysis were normal. Fasting blood glucose was 2.80 mmol/L (normal range: 3.90–6.10 mmol/L), and insulin was < 0.10 µIU/ml. Whole blood lactate was elevated at 5.00 mmol/L (normal range: 0.50–1.70 mmol/L), and pyruvate was 156.5 μmol/L (normal range: 20–100 μmol/L). Triglycerides were mildly elevated at 3.15 mmol/L (normal range: <1.70 mmol/L), while high-density lipoprotein, low-density lipoprotein, and uric acid levels were within normal limits. Urinalysis revealed 1 + ketones.

Abdominal ultrasound showed no evidence of hepatosplenomegaly.

## 3. Methods

### 3.1. Ethics statement

The study was approved by the Ethics Committee of Tongji Hospital, Tongji Medical College, Huazhong University of Science and Technology (Ethics Approval Number: TJ-IRB20180703). The patient and his parents provided written informed consent to participate in this study.

### 3.2. Gene mutation detection

Given the patient’s history of growth retardation, fasting hypoglycemia, hyperlactatemia, hyperpyruvicemia, and urine organic acid profile suggestive of lactic acidosis with ketosis, whole exome sequencing was performed after informed consent from the patient’s parents. Peripheral blood samples (4 mL) were collected from the proband and her parents, and genomic DNA was extracted using a standard salting-out method. Exome sequencing was conducted on the Illumina Hiseq X Ten platform, achieving an average depth of >150×, with 10 × coverage > 95% and 20 × coverage > 90%. Variant annotation was performed using databases including the 1000 Genomes Project, ESP6500, gnomAD, dbSNP, ClinVar, and HGMD. Variant pathogenicity was predicted using SIFT, Polyphen2, and MutationTaster, and classified according to the American College of Medical Genetics and Genomics guidelines.

### 3.3. Continuous glucose monitoring (CGM)

To assess blood glucose fluctuations, CGM was performed using a Medtronic iPro2 system. A sensor was inserted subcutaneously in the deltoid region on day 1. Blood glucose was measured every 5 minutes. Hypoglycemia (<3.90 mmol/L) and hyperglycemia (>7.80 mmol/L) were defined as per established criteria.^[4,5]^ The percentage of time spent in hypoglycemia or hyperglycemia was calculated as the proportion of readings within each range relative to the total monitoring duration.^[4,5]^

## 4. Result

### 4.1. Genetic testing results

**Figure 1.**
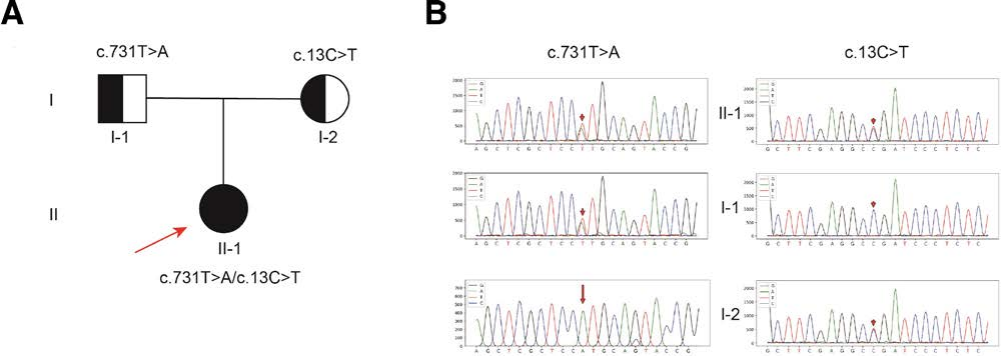
Family pedigree and Sanger sequencing results of the proband and both parents.

Whole exome sequencing identified 2 compound heterozygous mutations in the proband’s *GYS2* gene: c.13C > T (p.R5X) in exon 1, inherited from the mother, and c.731T > A (p.M244K) in exon 5, inherited from the father. Both mutations were classified as potentially pathogenic according to the American College of Medical Genetics and Genomics guidelines (2015) and validated by Sanger sequencing in the proband and her parents (Fig. 1).

### 4.2. CGM results

**Figure 2.**
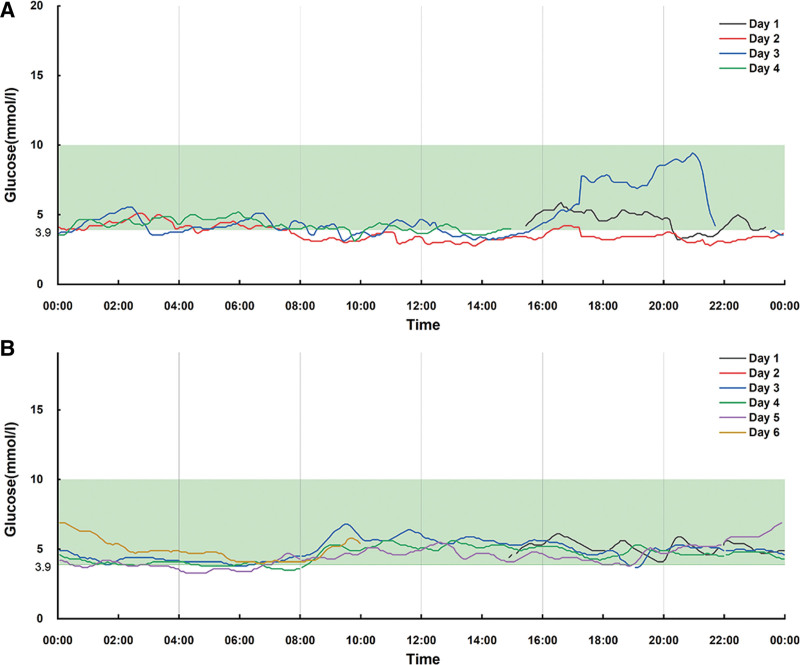
Continuous blood glucose monitoring before (A) and after (B) uncooked corn starch.

CGM over 4 days revealed recurrent hypoglycemia between 8:00 and 14:00, with blood glucose levels fluctuating between 2.78 mmol/L and 4.66 mmol/L (mean 3.60 ± 0.50 mmol/L). The lowest recorded blood glucose level was 2.78 mmol/L, and the frequency of hypoglycemic episodes (≤3.90 mmol/L) reached 25%. Additionally, the patient exhibited postprandial hyperglycemia between 18:00 and 22:00, with blood glucose levels averaging 5.92 ± 1.90 mmol/L and reaching a peak value of 9.44 mmol/L. Hyperglycemia (≥7.80 mmol/L) occurred in 10% of readings during this period (Fig. 2A).

### 4.3. Treatment

The patient determined the diagnosis of GSD0a by the clinical information and the results of genetic sequencing. To manage recurrent hypoglycemia, uncooked cornstarch therapy was initiated at a dose of 2 g/kg every 6 hours, administered between meals. CGM monitoring during the 6-day treatment period demonstrated improved glycemic control, with mean blood glucose increasing to 4.70 ± 0.70 mmol/L and hypoglycemia frequency decreasing to 12% (lowest value 3.30 mmol/L). No significant hyperglycemia was observed during the treatment period (Fig. 2B).

### 4.4. Follow-up

The patient was discharged and followed up every 3 months for a total of 3 years. During the follow-up period, growth parameters, fasting blood glucose, and relevant biochemical markers were monitored. The cornstarch dosage was adjusted between 1.5 g/kg and 2.0 g/kg based on the patient’s blood glucose levels.

**Figure 3.**
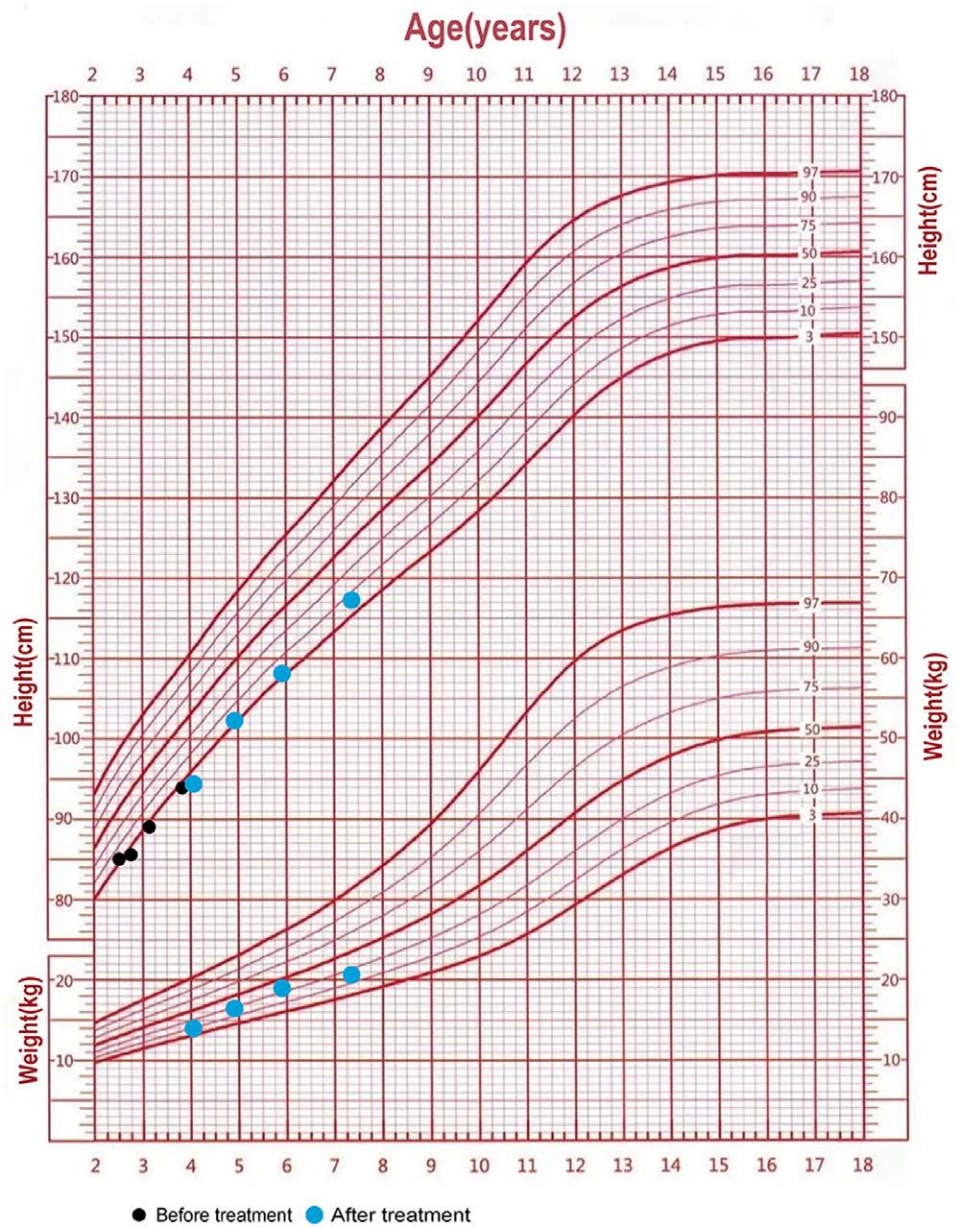
Growth chart of the patient before and after uncooked corn starch.

Before treatment, the patient’s growth velocity was 5.67 cm/year. Following treatment initiation, her growth velocity improved to 9.16 cm/year, 5.80 cm/year, and 7.20 cm/year at 1, 2, and 3 years, respectively. The patient’s height standard deviation score also improved from −2.24 before treatment to −1.82, −1.82, and −1.06 at each annual follow-up (Table 1). The slower growth rate in year 2 might be due to the patient’s inability to consistently obtain uncooked cornstarch during the coronavirus disease 2019 pandemic, leading to irregular treatment. Throughout the follow-up period, the patient’s weight remained between the 25th and 50th percentile (Fig. 3), with BMI values of 15.7, 16.2, and 14.6 at each follow-up (Table 1).

**Table 1:** Clinical data of the patient during the treatment.

Item	Baseline	3 months	1 year	2 years	3 years
HtSDS	−2.24	-	−1.82	−1.82	−1.06
Weight (kg)	14.0	14.5	16.5	19.0	20.0
BMI (kg/m^2^）	16.7	15.9	15.7	16.2	14.6
Growth velocity (cm/year)	5.67	-	9.16	5.80	7.20
Fasting blood glucose (mmol/L)	2.80	-	4.93	4.17	5.13
Lactic acid (mmol/L)	4.64	-	1.21	0.97	1.34
Pyruvate (µmol/L)	106.3	-	<30.0	<30.0	-
Triglycerides (mmol/L)	3.15	-	0.63	0.50	0.98
Cholesterol (mmol/L)	4.43	-	4.36	3.75	4.43
Uric acid (µmol/L)	279.1	239.0	223.8	275.2	233.0
HDL (mmol/L)	1.48	-	1.63	1.40	1.50
LDL (mmol/L)	1.98	-	2.68	2.19	2.48

BMI = body mass index, HDL = high-density lipoprotein, HtSDS = height standard deviation score, LDL = low-density lipoprotein.

During the 3-year course of cornstarch therapy, the patient maintained good fasting blood glucose control, with a minimum value of 5.13 mmol/L. No episodes of lactic acidosis, ketosis, or hyperlipidemia were observed, and the patient did not experience any neuropsychiatric symptoms such as somnolence or seizures. Liver function tests remained normal, and ultrasound examinations of the liver, kidneys, and heart revealed no significant abnormalities.

## 5. Discussion

Glycogen storage diseases (GSDs) are a group of inherited metabolic disorders caused by enzyme defects in glycogen synthesis or degradation. GSDs are categorized into more than 20 subtypes based on the specific enzyme deficiency, with GSD type I being the most prevalent. GSD type 0a is a relatively uncommon subtype caused by mutations in the *GYS2* gene, with an estimated incidence of <1 per million individuals. To date, only 33 cases have been reported, representing approximately 1% of all GSD cases.^[6]^

The *GYS2* gene, located on chromosome 12p12.1, encodes GS, a liver-specific enzyme that catalyzes the rate-limiting step in glycogen synthesis.^[7]^ GS facilitates the transfer of glucose from UDP-glucose to the terminal branch of glycogen via α-1,4 glycosidic bonds. Mutations in *GYS2* result in GS deficiency or dysfunction, impairing glycogen synthesis and disrupting glucose homeostasis. During fasting, inadequate liver glycogen stores lead to hypoglycemia. To compensate, the liver utilizes fatty acids for energy, resulting in excessive ketone body production and ketosis-induced hypoglycemia. Furthermore, GSD0a patients experience postprandial hyperglycemia due to the inefficient conversion of dietary glucose into liver glycogen. The excess glucose can be redirected to the glycolytic pathway, potentially causing lactic acidosis and hyperlipidemia.

To date, 37 pathogenic variants have been identified in the *GYS2* gene, including missense, nonsense, and deletion variants.^[6]^ The c.736C > T (p.R246*) variation is most common in the Turkish population (5/7 cases).^[2,6,8–11]^ Previous studies have not established a clear genotype–phenotype correlation. For example, individuals with the c.736C > T mutation can present with either short stature or normal height. In our study, 2 variants were detected: c.731T > A (p.M244K) and c.13C > T (p.R5X). The c.731T > A mutation was found in normal-height siblings by Liao et al, but they exhibited differing clinical presentations.^[12]^ The proband, a 4-year-old boy, experienced recurrent hypoglycemic seizures and language delay, while his sister only exhibited fasting hypoglycemia. Bachrach et al reported the c.13C > T variant in a 5-year-old boy with normal height and fasting hypoglycemia.^[11]^ Given the limited data on c.731T > A, we hypothesize that it may be a variant specific to the Chinese population.

Although fasting hypoglycemia and hepatomegaly are common in hepatic GSDs,^[13]^ clinical manifestations vary across subtypes. GSD0a patients typically experience hypoglycemia with symptoms like lethargy, pallor, and seizures. Compared to other types of GSD, the diagnosis of GSD0a is often delayed despite a similar median age of onset.^[6]^ Diagnosing GSD0a can be tricky for at least 3 reasons. First, the severity of hypoglycemia varies widely, with some patients exhibiting lethargy or seizures while others may remain asymptomatic. Second, GSD0a results from a defect in glycogen synthesis, and hepatomegaly is absent. Finally, GSD0a is not associated with elevated transaminase or creatine kinase levels. In our study, the patient initially presented only with short stature and lacked classic symptoms like hypoglycemia or liver abnormalities. This led to a diagnostic delay of over 1 year. Subsequent evaluation revealed fasting hypoglycemia, hyperlactatemia, ketosis, and a urinary organic acid profile consistent with lactic acidosis and ketosis. These findings raised suspicion for GSD0a, which was confirmed by genetic analysis.

CGM provides a more detailed assessment of glucose fluctuations compared to traditional self-monitoring of blood glucose, which often misses episodic hypoglycemia and dynamic glucose changes.^[14]^ White et al conducted a two-day CGM study on a 6-year-old male with GSD0a, observing that hyperglycemia predominantly occurred between 9:00–12:00 and 17:00–19:00.^[15]^ In our case, dynamic glucose monitoring revealed fasting hypoglycemia and postprandial hyperglycemia, with significant post-meal glucose increases observed only after dinner, unlike the elevations typically seen after breakfast or lunch in other reports. This variation in postprandial hyperglycemia may be associated with the patient’s specific glycogen synthase activity or dietary habits. Further investigation is needed to elucidate the underlying mechanisms. Nonetheless, the individual variability in glucose fluctuations highlights the utility of CGM in clinical management. CGM provides a comprehensive 24-hour glucose profile, aiding in the early detection of asymptomatic hypoglycemia and atypical hyperglycemia, thereby facilitating early diagnosis and adjustments in cornstarch therapy.

Short stature is a frequent clinical finding in patients with liver GSDs, yet the precise mechanisms contributing to this growth impairment remain elusive. In GSDIa and GSDXI, short stature is commonly observed. In GSD Ia, this may be attributed to disruptions in the growth hormone–insulin-like growth factor I axis. In GSD XI, short stature may result fromrecurrent chest infections, gastroenteritis, and proximal renal tubular dysfunction.^[16–19]^ In contrast, short stature is a less typical presentation in GSD0a, with very few cases reported where it is the sole initial symptom. Orho et al described a 4-year-old GSD0a patient with a height of 86.5 cm (<3rd percentile, −3 SD) and a weight of 11.4 kg (−3.00 SD) who showed improvement in fasting hypoglycemia with nighttime cornstarch therapy, though details on subsequent growth and development were not provided.^[2]^ Hacihamdioğlu et al reported a 5-year-old girl who presented with short stature: her initial height was 97.5 cm (−2.5 SDS), and after 1 year of treatment with 1 g/kg cornstarch, her height increased to 104.5 cm (−2.00 SDS).^[3]^ In our case, the patient initially presented with a height of −2.24 SDS and an annual growth rate of 5.67 cm/year. After diagnosis and initiation of cornstarch therapy, there was a marked improvement in height to −1.06 SDS. Throughout the treatment, the patient did not experience fasting hypoglycemia, with an average fasting blood glucose of 5.13 mmol/L, nor were there any episodes of lactic acidosis, ketosis, or neuropsychiatric symptoms such as somnolence or seizures.

## 6. Conclusion

In conclusion, our case report underscores the importance of considering GSD0a in the differential diagnosis of children presenting with isolated short stature, even in the absence of fasting hypoglycemia or hepatomegaly. This study also demonstrates the value of CGM in identifying asymptomatic hypoglycemia and atypical postprandial hyperglycemia, enabling personalized management of GSD0a patients. Early diagnosis and timely initiation of cornstarch therapy can significantly improve growth outcomes and prevent potential complications associated with GSD0a.

## Acknowledgments

We thank the patient and her family for their participation in this study.

## Author contributions

**Conceptualization:** Yan Liang.

**Data curation:** Hao Fu, Aoyu Yang.

**Supervision:** Yan Liang.

**Writing – original draft:** Hao Fu.

**Writing – review & editing:** Hao Fu, Caiqi Du, Yan Liang.
